# Soluble urokinase plasminogen activator receptor level in individuals of advanced age

**DOI:** 10.1038/s41598-020-72377-w

**Published:** 2020-09-22

**Authors:** Rafal N. Wlazel, Katarzyna Szwabe, Agnieszka Guligowska, Tomasz Kostka

**Affiliations:** 1 Department of Laboratory Diagnostics and Clinical Biochemistry, Medical University of Lodz, Lodz, Poland; 2 Department of Geriatrics, Medical University of Lodz, Lodz, Poland

**Keywords:** Immunochemistry, Proteins, Biomarkers, Risk factors

## Abstract

Soluble urokinase plasminogen activator receptor (suPAR) is a biomarker whose clinical value has been tested in various groups of patients. The aim of the present study was to determine the suPAR level in a previously uninvestigated population of 182, generally healthy, community-dwelling seniors aged 74–89 years. In addition to suPAR level, selected laboratory parameters of heart and kidney function, lipid and C-reactive protein levels were determined. A group of 45 younger individuals aged 24–66 years was used for comparison. The seniors had higher suPAR levels than younger controls: 3.79 ng/mL (95% CI 3.64–3.96 ng/mL) vs. 3.16 ng/mL (95% CI 2.86–3.45 ng/mL). These levels increased further with advancing age, and were similar in women and men. A multiple regression model confirmed that biomarker level was related to cardiac function, renal function and inflammation, and this remained after adjusting for age. These correlation patterns were similar in older women and men.

## Introduction

The soluble urokinase plasminogen activator receptor (suPAR) is a useful biomarker that reflects immune activation in an individual and is known to be involved in the inflammation process^1^. Its elevated levels have been found to predict morbidity and mortality across acute and chronic diseases in the general population. One of the largest such cohort studies found suPAR level to correlate with the development of cardiovascular diseases, cancer and premature mortality in a population in Denmark^2^. It has also been found that suPAR level is strongly related to lifestyle. Conditions associated with low-grade chronic inflammation and subclinical organ damage, such as daily smoking, obesity, unhealthy diet, physical inactivity, elevated low-density lipoprotein cholesterol level and a history of cardiovascular disease, are known to coexist with an elevated suPAR level, despite being independent of other inflammation biomarkers such as C-reactive protein (CRP)^3,4^.

One non-modifiable risk factor that correlates with an increase of suPAR level is age. Although studies have examined the suPAR level in different populations of patients, none of the non-acute care individuals included in the study were older than 68 years of age (median age 65.5), at the time of blood collection^2,5^. However, recent studies evaluating the use of suPAR in short-term risk prediction strategies for acute medical patients have been based around subgroups of patients with a median age of 61.3 (43.3–76.3) years^6^ or 80.7 ± 7.4 years^7^.

Aging is associated with a variety of immunological responses activated by biological and stress factors and natural hormonal changes, resulting in a gradual decline in organ function^8^. Systematic alterations in cardiac and vascular structure and function lead to heart failure and cerebrocardiovascular events, while changes in glomeruli result in a decline in renal function. The presence of low-grade chronic inflammation processes, mainly on the endothelial level, results in subclinical organ damage leading to diabetes, heart failure, malignancy and inflammatory systemic diseases^9^. Those processes are self-accelerating and together with malnutrition, another ageing-related problem, they increase the risk of morbidity and mortality. Many of the biochemical pathways associated with ageing, including immune response, cell migration and adhesion, angiogenesis, proliferation and chemotaxis, involve the activity of the suPAR protein, whose level is directly associated with chronic inflammation^1^. Its level reflects the intensification of actual immunological defense processes of the individual and the potential risk associated with them, and hence the theory of ageing processes.

As the proportion of the population above age 65 grows, so too does the need for health care among that population, and the need for accurate diagnoses and prognoses. One of the greatest challenges for laboratory medicine in this field is to provide adequate reference intervals or any relative information about the interpretation of laboratory parameters in the elderly. This aspect is further complicated by the problem surrounding the definition of health in the elderly^10^. Greater studies are needed of the properties of suPAR in older healthy individuals as these constitute the majority of acute medical patients.

The value of suPAR as a prognostic biomarker is typically realized by point-of-care test devices (suPARnostic Quick Triage) based on the lateral-flow immunoassay principle: these being mostly used for initial triage purposes in emergency departments. A semi-automated assay (suPARnostic ELISA AUTO Flex ELISA) has also been routinely used in the Danish Hvidorve Hospital. Although a fully-automated immunoturbidimetric assay for quantitative suPAR measurement (suPARnostic TurbiLatex) has been available for more than year now, no robust reference intervals are known, and there are no data about biological variation of suPAR. Likewise, the studies concerning the clinical value of the biomarker focus mostly on estimating cut-off values for the prediction of adverse outcomes in various groups of patients and rely on previously and still available ELISA kit (suPARnostic ELISA).

### Aims

The aim of this study was to determine the suPAR level in a generally healthy non-disabled older population. As a clear definition of health in seniors is problematic, the present study examines the relationships of suPAR and other laboratory markers and certain comorbidities affecting immune activation, to the analogous extent that it concerns younger individuals.

## Methods

### The study population

The study population was composed solely of Caucasian residents of Central Poland. All were patients admitted to the Geriatrics Outpatient Clinic of the University Clinical Hospital in Lodz. Over the period March 2017 to January 2018, out of a total of 326 consecutive patients, 182 generally healthy, independent, community-dwelling individuals aged 74–88 years (median 79 years) were enrolled.

The exclusion criteria were based on current knowledge about the independent factors associated with elevated suPAR level: current acute inflammation process (CRP > 5 mg/L), daily smoking, end-stage renal disease (Stage > G3a in GFR category or dialysis), autoimmune diseases, immunosuppressive treatment, active malignancy and metastatic cancer, heart failure > II NYHA (New York Heart Association^11^) stage, impaired cognitive function (Mini-Mental State Examination^12^ < 15 scores), life expectancy < 6 month. To exclude any potential bias, 45 younger individuals aged 24–66 years (median 46) were included as a comparative group: they were examined during the same period in the Central Medical Laboratory, University Clinical Hospital in Lodz. The same exclusion criteria were applied as those described above, except the Mini-Mental State Examination. The number of individuals in this group was equally balanced across every 10-year age range.

### Laboratory measurements

Laboratory parameters were analyzed in serum samples taken after fasting. After centrifugation, part of the serum was immediately aliquoted and frozen at – 80 °C for up to 10 months. For the suPAR analysis, the samples were thawed, thoroughly mixed and centrifuged, and their suPAR concentrations were assessed using suPARnostic ELISA (Virogates, Denmark). To minimize analytical variation, all the samples were analyzed in duplicate during a single run, using a single lot of reagent and by single analyst. The imprecision of the assay varied from 1.7% for the highest mean concentration to 4.7% for the lowest.

The following laboratory tests were performed on native samples within one hour of blood collection: total cholesterol (TC), high-density lipoprotein cholesterol (HDL-C), low-density lipoprotein cholesterol (LDL-C), triglycerides (TG), creatinine (CREA), C-reactive protein (CRP), glycated hemoglobin (HbA1c) were measured using the AU680 analyzer and commercially-available assays (Beckman Coulter, Brea, USA), while troponin T (TnT) and N-terminal pro-B type natriuretic peptide (NT-proBNP), were determined by electrochemiluminescence immunoassay with a Cobas e611 analyzer (Roche Diagnostics, Rotkreutz, Switzerland). For CRP and TnT, highly-sensitive methods were applied. Glomerular filtration rate (GFR) was calculated using the Chronic Kidney Disease – Epidemiology Collaboration formula^13^.

### Statistical analyses

The Shapiro–Wilk test was used to assess the normality of distribution for the investigated parameters. As they were found to differ significantly from normality, the results and baseline characteristics are presented as median and interquartile range for continuous variables, and as percentages for categorical variables. Tukey’s Box-and-Whisker plot was used to exclude outliers of the suPAR concentration prior to further analysis^14^. Differences between groups were tested with the use of the Mann–Whitney *U* test, and Spearman’s rank correlation analysis was performed to analyze correlations between tested parameters. Multiple regression analysis was applied to adjust for age and select factors that independently predict the suPAR level. For the multivariable analyses, the data were log-transformed to improve their adherence to a normal distribution. The statistical analysis was performed using MedCalc Statistical Software version 17.5.5 (MedCalc Software bvba, Ostend, Belgium; https://www.medcalc.org; 2017). A significance level of α = 0.05 was used in all tests.

### Compliance with ethical standards

The study was approved by the Bioethics Committee of Medical University of Lodz in accordance with the Declaration of Helsinki (ref. RNN/647/14/KB). All patients gave written consent for participation in the study.

## Results

**Figure 1:**
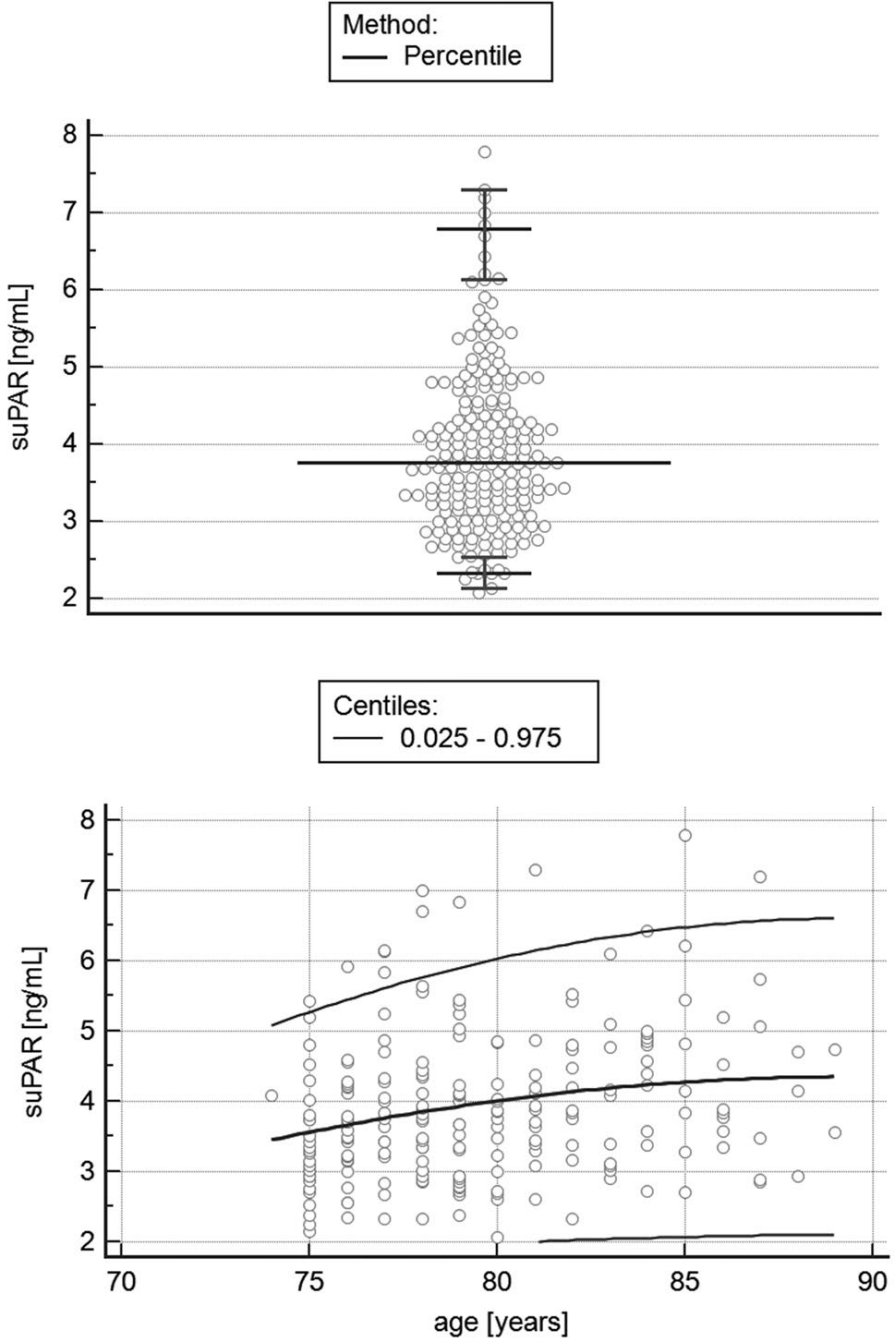
The distribution of the suPAR concentration in the study group after excluding outliers (upper part). The 95% percentile interval (with 90% confidence interval for lower and upper limit) following the CLSI guidelines C28-A3 is presented, also including age-related distribution (lower part).

The sake of clarity, all the numerical values describing suPAR concentration are given in ng/mL. After excluding three outliers (individuals with suPAR concentrations 9.34, 9.50 and 10.58) according to the *CLSI C28-A3* protocol “*Defining, Establishing, and Verifying Reference Intervals in the Clinical Laboratory; Approved Guideline*”, the summary statistics of the marker were as follow: suPAR values were found to range from 2.07 to 7.79, with a median of 3.79 and interquartile range 3.17–4.52. The 95% interval of the results, established with non-parametric percentile method (*CLSI C28-A3*), ranged from 2.33 (90% CI 2.14–2.15) to 6.79 (90% CI 6.13–7.30) ng/mL (Fig. 1). The baseline characteristics of the study population are presented in Table 1.

**Table 1:** Baseline characteristics of the studied population, divided into a seniors group and a comparison group of younger individuals.

Variable	Study group (seniors) n = 182	Reference group (younger individuals) n = 45	Significance of the difference
Age (years)	79 (77–82)	46 (36–60)	P < 0.0001
Sex (females)	126 (69%)	29 (64%)	P = 0.537
Statin treatment	78 (43%)	5 (11%)	P = 0.0001
suPAR (ng/mL)	3.79 (3.17–4.52)	3.16 (2.65–3.51)	P < 0.0001
TC (mmol/L)	5.46 (4.66–6.35)	5.41 (4.92–5.84)	P = 0.617
HDL-C (mmol/L)	1.63 (1.35–1.93)	1.45 (1.28–1.72)	P = 0.011
LDL-C (mmol/L)	3.21 (2.40–3.93)	3.63 (2.85–4.50)	P = 0.005
TG (mmol/L)	1.31 (1.03–1.63)	1.35 (0.99–1.97)	P = 0.421
HbA1c (%)	5.6 (5.3–5.9)	5.0 (4.8–5.2)	P < 0.0001
CREA (µmol/L)	75 (82–104)	85 (74–95)	P = 0.004
eGFR CKD-EPI (mL/min/1.73m^2^)	73 (61–81)	77 (71–92)	P < 0.0001
CRP-hs (mg/L)	1.61 (0.95–2.94)	1.10 (0.52–2.21)	P = 0.003
TnT-hs (ng/L)	9.3 (8.3–10.1)	5.5 (4.6–6.5)	P < 0.0001
NT-proBNP (pg/mL)	212 (108–420)	29 (20–42)	P < 0.0001

The variables are expressed as median and interquartile range.

Significant differences in suPAR concentration were observed between the study and the control groups, with median values of 3.79 (95% CI 3.45–3.96) for the study group and 3.16 (95% CI 2.86–3.45) for the controls (p < 0.0001). The suPAR concentration was higher in the studied group of the geriatric patients in comparison to the younger controls and the 95% CIs for the medians did not overlap. Regarding the non-modifiable risk factors, a correlation was also observed between biomarker level and age across the studied group (r_S_ = 0.26; p = 0.0005). However, the sex-related differences in suPAR level, reported in previous studies, were not observed in our population of seniors. In contrast, a noticeable difference was observed among the younger participants, with median suPAR level being significantly higher in women (median 3.31; CI 3.12–3.54) than in men (median 2.65; CI 2.36–3.34) (p = 0.011).

The study group was further divided into two subgroups according to median age (Table 2). Among those observed in the younger population associations between suPAR and laboratory parameters, only correlation between the protein and creatinine, estimated GFR, TnT, NT-proBNP and CRP level were observed in the studied group. suPAR level did not correlate with lipid level, nor with the indicator of average glucose level. In addition, all parameters apart from CRP were found to differ significantly between the two age-related subgroups. The suPAR level was also higher in the subgroup of older geriatric patients.

**Table 2:** Detailed characteristics of the studied group divided into two subgroups, based on a median age of 79 years.

Variable	Age ≤ 79 years n = 103	Age > 79 years n = 79	Significance of the difference	Correlation with suPAR in the entire studied population
Sex (females)	83 (80%)	46 (58%)	P = 0.743	N/A
suPAR (ng/mL)	3.67 (3.01–4.26)	4.15 (3.40–4.87)	P = 0.001	N/A
TC (mmol/L))	5.21 (4.53–6.32)	5.26 (4.52–6.12)	P = 0.77	r_S_ = − 0.081; P = 0.21
HDL-C (mmol/L)	1.53 (1.32–1.76)	1.61 (1.34–1.84)	P = 0.47	r_S_ = 0.015; P = 0.80
LDL-C (mmol/L)	3.00 (2.51–3.87)	3.06 (2.43–3.83)	P = 0.95	r_S_ = 0.077; P = 0.21
TG (mmol/L)	1.31 (1.01–1.65)	1.23 (0.93–1.52)	P = 0.06	r_S_ = 0.070; P = 0.32
HbA1c (%)	5.5 (5.3–6.0)	5.6 (5.4–5.8)	P = 0.85	r_S_ = − 0.092; P = 0.28
CREA (µmol/L)	75 (65–86)	79 (69–95)	P = 0.03	r_S_ = 0.320; P < 0.001
eGFR CKD-EPI (mL/min/1.73 m^2^)	74 (63–84)	69 (53–79)	P = 0.0006	r_S_ = − 0.334; P < 0.001
CRP-hs (mg/L)	1.68 (0.95–3.13)	1.58 (0.97–2.66)	P = 0.48	r_S_ = 0.186; P = 0.06
TnT-hs (ng/L)	8.2 (5.6–11.7)	11.6 (8.2–16.1)	P < 0.001	r_S_ = 0.298; P < 0.001
NT-proBNP (pg/mL)	154 (96–285)	300 (159–678)	P < 0.001	r_S_ = 0.289; P < 0.001

The numeric values of the measurands are expressed as median and interquartile range.

*N/A* not applicable, *r*_*S*_ Spearman correlation coefficient.

All the above correlations remained statistically significant after adjusting for age in multiple regression analysis. The model used as covariates parameters that significantly correlated with suPAR level as univariate associations. Age, CRP, TnT, NT-proBNP and eGFR fit the model and appeared to contribute significantly to the prediction of suPAR level, with the total significance of the model p < 0.0001 (Table 3).

**Table 3:** The impact of selected variables on suPAR concentration: a regression model taking into account statistically relevant correlations with suPAR protein level.

	Independent variable	Adjusted R^2^	F-ratio	Coefficient ß ± SE	P value
suPAR (ng/mL)		0.17	15.01		< 0.0001
	Age (years)			0.041 ± 0.0206	0.046
	CRP-hs (mg/L)			0.042 ± 0.0193	0.030
	TnT-hs (ng/L)			0.021 ± 0.0092	0.027
	NT-proBNP (pg/mL)			0.00048 ± 0.00014	0.001
	eGFR CKD-EPI (mL/min/1.73 m^2^)			− 0.021 ± 0.0054	0.0001

The described above correlations were also noticeable in the gender subgroups (Table 4). The suPAR level strongly correlated with age, CRP and biomarkers associated with heart and kidney condition in the female subgroup. Weaker correlations were visible in the male subgroup: significance was borderline for the kidney-related biomarkers and the TnT, and the suPAR level did not correlate significantly with CRP. This may be explained by the smaller size of the male group. As the subgroups were homogenous in terms of age, this covariance could not have had a significant influence on the relationships. Finally, the similarity between the two groups was further confirmed by the observed homogeneity between the regression slopes (p > 0.05).

**Table 4:** Detailed characteristics of the studied group divided into two subgroups according to sex.

Variable	Women, 69% n = 126	Correlation with suPAR in women	Men, 31% n = 56	Correlation with suPAR in men	Significance of the difference (women vs men)
Age (years)	79 (77–82)	r_S_ = 0.255 p = 0.0019	79 (76–82)	r_S_ = 0.275 p = 0.032	P = 0.988
suPAR (ng/mL)	3.76 (3.15–4.45)	N/A	3.8 (3.27–4.63)	N/A	P = 0.386
TC (mmol/L))	5.50 (4.80–6.42)	r_S_ = − 0.039 P = 0.640	4.62 (4.01–5.65)	r_S_ = − 0.126 P = 0.320	P < 0.0001
HDL-C (mmol/L)	1.66 (1.40–1.90)	r_S_ = − 0.033 P = 0.695	1.36 (1.11–1.52)	r_S_ = 0.210 P = 0.095	P < 0.0001
LDL-C (mmol/L)	3.21 (2.64–4.07)	r_S_ = − 0.023 P = 0.784	2.64 (2.20–3.65)	r_S_ = − 0.146 P = 0.255	P = 0.0005
TG (mmol/L)	1.26 (1.00–1.64)	r_S_ = − 0.042 P = 0.617	1.30 (0.89–1.64)	r_S_ = − 0.140 P = 0.268	P = 0.76
HbA1c (%)	5.5 (5.3–5.9)	r_S_ = − 0.135 P = 0.103	5.6 (5.4–5.9)	r_S_ = 0.003 P = 0.978	P = 0.291
CREA (µmol/L)	70 (63–84)	r_S_ = 0.364 p = < 0.0001	90 (78–100)	r_S_ = 0.257 p = 0.078	P < 0.0001
eGFR CKD-EPI (mL/min/1.73 m^2^)	72 (59–82)	r_S_ = − 0.395 P < 0.0001	73 (64–81)	r_S_ = − 0.259 P = 0.075	P = 0.568
CRP-hs (mg/L)	1.69 (0.98–2.86)	r_S_ = 0.205 P = 0.013	1.38 (0.81–3.28)	r_S_ = 0.204 P = 0.143	P = 0.418
TnT-hs (ng/L)	8.2 (5.5–11.8)	r_S_ = 0.325 P = 0.0001	12.1 (8.3–15.2)	r_S_ = 0.258 P = 0.062	P < 0.0001
NT-proBNP (pg/mL)	219 (119–401)	r_S_ = 0.345 P < 0.0001	161 (96–440)	r_S_ = 0.329 P = 0.009	P = 0.559

The measurands are expressed as mean and interquartile range.

*N/A* not applicable, *r*_*S*_ Spearman correlation coefficient.

In addition, none of the patients with hypertension (74%, n = 134), diabetes (48%, n = 87), coronary artery disease (CAD) (6%, n = 11) or history of any cerebrocardiovascular events (12%, n = 23) displayed any significantly higher suPAR level (p > 0.05). Likewise, former smokers (38%, n = 69) did not have higher suPAR level, medians: 3.79 vs 3.76; p = 0.967.

## Discussion

The need to provide greater medical assistance of an aging population that requires medical assistance is currently hampered by the lack of robust information concerning laboratory parameters in Geriatrics. In addition, no previous study has comprehensively explored the issue of suPAR level in a population of advanced age, although many have suggested that suPAR levels increase with age^1–3^: a correlation also confirmed in our present study. Not only was a significantly higher protein level observed in the group of geriatric patients compared the younger individuals, but variation was also observed among the geriatric group.

Interestingly, while previous studies indicate that women tend to demonstrate a higher suPAR level than men, no such difference was observed in the present group. This may be related to the relatively worse general physical condition of the men; in addition, the present study included twice as many women as men, which is to be expected, considering the generally-observed tendency of women to live longer.

It was previously proved that suPAR level is elevated in individuals with the history of cardiovascular diseases and their complications. It also predicts adverse cardiac events, especially in patients after the first episode^15,16^. The geriatric population in our present study consisted of patients with no or only mild symptoms of heart failure, classed as NYHA I or II. It was not possible to exclude all the patients with any history of CAD and its risk factors. Nevertheless, the existence of previous hypertension, diabetes or cerebrocardiovascular events did not differentiate the study population with regard to suPAR level. This may indicate that the protein represents a potential risk factor in older patients, independent of coexisting life-threatening comorbidities. It is also possible that these associations might become detectable in more diseased than our older population.

Regarding the laboratory parameters examined in this study, suPAR level was strongly correlated with biomarkers of heart failure and necrosis of myocardium cells. NT-proBNP and TnT were also higher in the older subgroup of the geriatric patients. These findings are in accordance with those of Eggers et al.^17^, who report the presence of elevated levels of those biomarkers in different patterns of CVD among elderly participants; they also suggest that these levels may reflect changes in cardiovascular risk profile which might be modifiable.

suPAR level was also found to strongly correlate with laboratory markers of kidney function. This observation is in accordance with the observation that in chronic kidney disease (CKD) the biomarker correlates with reduced GFR^18,19^. The present study excluded patients with CKD at stages greater than 3a. Nevertheless, suPAR level was found to strongly correlate with creatinine concentration and GFR across the studied group, which is in accordance with previous studies^2–4,18,19^. We are aware that the CKD-EPI formula was not standardized for the population of seniors^13^; however, we used this formula, as currently recommended, to remain comparability with other studies concerning suPAR.

A good correlation was also observed between suPAR and CRP in our patients and this relationship has also been frequently mentioned in previous studies^2–5,15–19^. Nevertheless, the p-values of our present findings were more robust for suPAR. Surprisingly, no correlation was observed between the biomarker and TC, nor LDL-C concentrations in the studied group of patients: the most probable reason for this is that 43% of the patients were undergoing long-term statin treatment. In addition, current daily smokers were excluded from the study, with only former smokers being included. Although there is evidence that smoking is the strongest lifestyle factor associated with elevated suPAR level^3^, there are studies showing that its level falls after smoking cessation^20,21^. Similarly, the former smokers included in the present study did not demonstrate any higher biomarker level.

Due to the varied (patho)physiology of aging, it is difficult to provide an adequate frame of reference for the laboratory test results. A major obstacle is the definition of health in seniors, because it is extremely rare to find individuals without any medical condition or medication in older adulthood. As suPAR level appears to have more prognostic than diagnostic power, its analysis is better employed as part of a strategy of providing cut-off or decision levels, as is noticeable across previous publications considering the biomarker. Hence, rather than attempting to provide clear reference intervals, the aim of the present study was to determine the suPAR level in an older population. Our results are in line with previous findings about the biomarker, extrapolating the observed in younger individuals correlations to the population of seniors. All noticed correlations indicated that age, the intensification of the inflammation, measured with CRP, and the actual heart efficiency, reflected by NT-proBNP concentration, influenced suPAR level. Although these correlations were clearer among women, probably due to the greater size of this group, similar general tendencies are also visible among men; in addition, both groups demonstrated homogenous regression slopes. Therefore, it may be possible that suPAR is also an independent prognostic biomarker in advanced age, independent of comorbidities. This finding is in accordance with Shultz et al.^7^, who report that while short-time risk scoring in elderly patients differs from that in younger adults in an Emergency Department setting, no such difference is observed for suPAR, which was not significantly worse in the elderly. However, further follow-up studies on quality of life, morbidity and mortality are needed before drawing any firm conclusions regarding the usefulness of the protein in long-time risk stratification in geriatric patients. This will also make it possible to establish a reliable right-sided upper reference limit that could be a decisive value for risk stratification in the elderly population.

## Conclusion

suPAR level is higher in the population aged ≥ 74 years than on one composed of middle-age individuals, and it increases with advancing age. The biomarker may also be an independent candidate for risk stratification in older patients, bearing in mind its potential to directly reflect immune activation and subclinical organ damage; however, further prospective studies are needed to confirm this.

## Data Availability

All data obtained in this study are presented in the manuscript. All datasets are available from the corresponding author on reasonable request.
